# Bromido(dodecafluorosubphthalo­cyaninato)boron(III)

**DOI:** 10.1107/S1600536810041863

**Published:** 2010-11-06

**Authors:** Graham E. Morse, Jozef F. Maka, Alan J. Lough, Timothy P. Bender

**Affiliations:** a Department of Chemical Engineering & Applied Chemistry, University of Toronto, 200 College Street, Toronto, Ontario, Canada M5S 3E5; b Department of Chemistry, University of Toronto, 80 St. George Street, Toronto, Ontario, Canada M5S 3H6

## Abstract

The title compound, C_24_BBrF_12_N_6_ or Br-F_12_BsubPc (BsubPc is boronsubphtalocyanine), has a bowl-shaped structure with an approximate mol­ecular *C*_3*v*_ symmetry characteristic of boronsubphthalocyanine compounds. In the crystal, mol­ecules are arranged in one-dimensional columns and the boron–subphthalocyanine units within each column are offset and angled in a bowl-to-ligand packing arrangement such that the axial Br atom rests in the aromatic concaved bowl of the neighboring subphthalocyanine with an inter­molecular Br⋯B distance of 3.721 (3) Å.

## Related literature

For general background to boronsubphthalocyanines, see: Claessens *et al.* (2002 ▶). For examples of related halogenated boronsubphthalocyanines, see: Morse *et al.* (2010 ▶); Paton *et al.* (2010 ▶); Rodriguez-Morgade *et al.* (2008 ▶); Sharman & van Lier (2005 ▶); Ros-Lis *et al.* (2005 ▶); Fuduka *et al.* (2002 ▶); Claessens & Torres (2002 ▶). For applications of boronsubphthalocyanines in organic electronics, see: Mutolo *et al.* (2006 ▶); Gommans *et al.* (2007 ▶, 2009 ▶); Kumar *et al.* (2009 ▶); Ma *et al.* (2009*a* ▶,*b* ▶); Klaus *et al.* (2009 ▶); Chen *et al.* (2009 ▶, 2010 ▶); Díaz *et al.* (2007 ▶); Yasuda & Tsutsui (2007 ▶); Renshaw *et al.* (2010 ▶). For van der Waals radii, see: Bondi (1964 ▶).
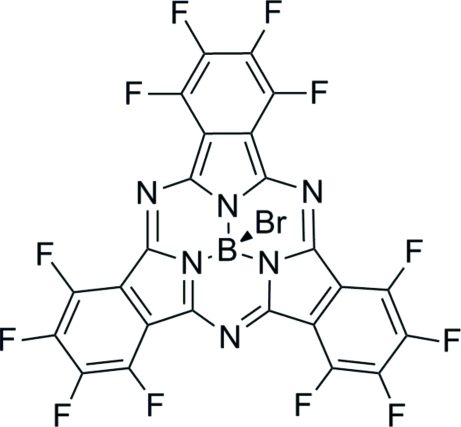


## Experimental

### 

#### Crystal data

C_24_BBrF_12_N_6_
*M* *_r_* = 691.02
Monoclinic, 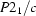

*a* = 11.1681 (5) Å
*b* = 10.8858 (2) Å
*c* = 19.0664 (7) Å
β = 95.2270 (15)°
*V* = 2308.33 (14) Å^3^
*Z* = 4
Mo *K*α radiation
μ = 1.91 mm^−1^
*T* = 150 K
0.14 × 0.14 × 0.10 mm

#### Data collection

Nonius KappaCCD diffractometer
Absorption correction: multi-scan (*SORTAV*; Blessing, 1995 ▶) *T*_min_ = 0.775, *T*_max_ = 0.835
15166 measured reflections
5263 independent reflections
3728 reflections with *I* > 2σ(*I*)
*R*_int_ = 0.046

#### Refinement

*R*[*F* ^2^ > 2σ(*F* ^2^)] = 0.045
*wR*(*F* ^2^) = 0.109
*S* = 1.04
5263 reflections
397 parameters
Δρ_max_ = 0.95 e Å^−3^
Δρ_min_ = −0.41 e Å^−3^

### 

Data collection: *COLLECT* (Nonius, 2002 ▶); cell refinement: *DENZO-SMN* (Otwinowski & Minor, 1997 ▶); data reduction: *DENZO-SMN*; program(s) used to solve structure: *SIR92* (Altomare *et al.*, 1994 ▶); program(s) used to refine structure: *SHELXTL* (Sheldrick, 2008 ▶); molecular graphics: *PLATON* (Spek, 2009 ▶) and *Mercury* (Macrae *et al.*, 2008 ▶); software used to prepare material for publication: *SHELXTL*.

## Supplementary Material

Crystal structure: contains datablocks global, I. DOI: 10.1107/S1600536810041863/nc2200sup1.cif

Structure factors: contains datablocks I. DOI: 10.1107/S1600536810041863/nc2200Isup2.hkl

Additional supplementary materials:  crystallographic information; 3D view; checkCIF report

## Figures and Tables

**Fig. 1.**
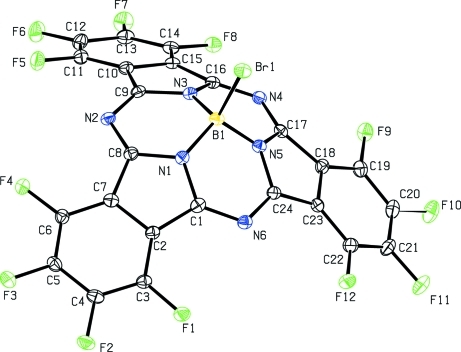
The molecular structure of the title compound with 30% probability ellipsoids.

**Fig. 2.**
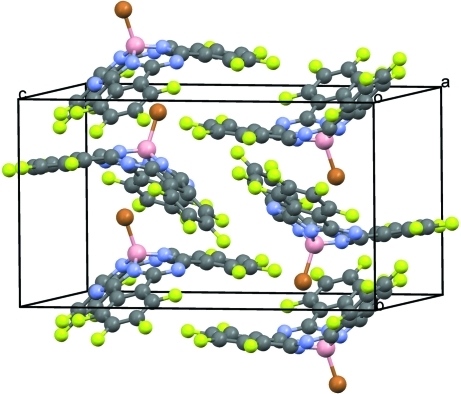
Part of the crystal structure of the title compound.

## supplementary crystallographic information

### Comment

Boronsubphthalocyanine (BsubPc), a lower analogue of phthalocyanine, is of interest to researchers in the field of organic electronics (Morse *et al.*, 2010; Mutolo *et al.* 2006; Gommans *et al.* 2007; Gommans *et al.* 2009; Kumar *et al.* 2009; Ma *et al.* 2009*a*; Klaus *et al.* 2009; Ma *et al.* 2009*b*; Chen *et al.* 2010; Chen *et al.* 2009; Díaz *et al.* 2007; Yasuda *et al.* 2007; and Renshaw *et al.* 2010). We have synthesized the title compound as it is a precursor to fluorinated phenoxy-BsubPcs (Morse *et al.* 2010; Paton *et al.* 2010). The molecular structure of the title compound is shown in Fig. 1. In the crystal structure the molecules are arranged in a concave bowl to ligand motif similar to those of F-F_12_BsubPc (Rodriguez-Morgade *et al.* 2008) and Cl-F_12_BsubPc (Fuduka *et al.* 2002) whereby the axial halogen atom lies within the concaved face of the BsubPc molecular fragment in close proximately to the boron atom. The net effect is the formation of distinctive columns throughout the crystal structure (Fig. 2). In this arrangment the intermolecular bromine to nitrogen distances are 3.420 (2), 3.466 (2), and 3.427 (2) Å which are close to the sum of the van der Waals radii at 3.40 Å (1.85 Å[Br]+1.55 Å[N]). The distance between Br1 and B1(1 - *x*, *y* + 1/2, -*z* + 1/2) is 3.721 (3) Å which is less the sum of the van der Waals radii at 3.85 Å (1.85 Å[Br]+2.0 Å[B]). The axial boron-bromine bond is oriented towards the inner 5-membered ring of the nieghboring BsubPc unit. This interaction occurs at a distance of 3.471 Å, less than the sum of the van der Waals raddi at 3.55Å (1.85 Å[Br]+1.70 Å[C]). The other two 5-membered rings are seperated from the bromine atom by a distance of 3.572 Å and 3.518 Å, near the sum of the respective van der Waals radii. Neighboring BsubPc units are separated by a B···B distance of 5.471 (5) Å. All van der Waals radii were calculated using the values determined by Bondi (1964).

### Experimental

Br-F_12_BsubPc was synthesized as previously reported (Morse *et al.* 2010). Single crystals suitable for X-ray diffraction were prepared by slow vapour diffusion of heptane into a solution of Br-F_12_BsubPc in benzene.

### Crystal data

**Table:**

C_24_BBrF_12_N_6_	*F*(000) = 1336
*M**_r_* = 691.02	*D*_x_ = 1.988 Mg m^−^^3^
Monoclinic, *P*2_1_/*c*	Mo *K*α radiation, λ = 0.71073 Å
Hall symbol: -P 2ybc	Cell parameters from 15166 reflections
*a* = 11.1681 (5) Å	θ = 2.6–27.5°
*b* = 10.8858 (2) Å	µ = 1.91 mm^−^^1^
*c* = 19.0664 (7) Å	*T* = 150 K
β = 95.2270 (15)°	Block, purple
*V* = 2308.33 (14) Å^3^	0.14 × 0.14 × 0.10 mm
*Z* = 4	

### Data collection

**Table:**

Nonius KappaCCD diffractometer	5263 independent reflections
Radiation source: fine-focus sealed tube	3728 reflections with *I* > 2σ(*I*)
graphite	*R*_int_ = 0.046
Detector resolution: 9 pixels mm^-1^	θ_max_ = 27.5°, θ_min_ = 2.6°
φ scans and ω scans with κ offsets	*h* = −14→14
Absorption correction: multi-scan (*SORTAV*; Blessing, 1995)	*k* = −12→14
*T*_min_ = 0.775, *T*_max_ = 0.835	*l* = −21→24
15166 measured reflections	

### Refinement

**Table:**

Refinement on *F*^2^	0 restraints
Least-squares matrix: full	Primary atom site location: structure-invariant direct methods
*R*[*F*^2^ > 2σ(*F*^2^)] = 0.045	Secondary atom site location: difference Fourier map
*wR*(*F*^2^) = 0.109	*w* = 1/[σ^2^(*F*_o_^2^) + (0.0553*P*)^2^] where *P* = (*F*_o_^2^ + 2*F*_c_^2^)/3
*S* = 1.04	(Δ/σ)_max_ = 0.001
5263 reflections	Δρ_max_ = 0.95 e Å^−^^3^
397 parameters	Δρ_min_ = −0.41 e Å^−^^3^

### Special details

**Table:**

Geometry. All e.s.d.'s (except the e.s.d. in the dihedral angle between two l.s. planes) are estimated using the full covariance matrix. The cell e.s.d.'s are taken into account individually in the estimation of e.s.d.'s in distances, angles and torsion angles; correlations between e.s.d.'s in cell parameters are only used when they are defined by crystal symmetry. An approximate (isotropic) treatment of cell e.s.d.'s is used for estimating e.s.d.'s involving l.s. planes.
Refinement. Refinement of *F*^2^ against ALL reflections. The weighted *R*-factor *wR* and goodness of fit *S* are based on *F*^2^, conventional *R*-factors *R* are based on *F*, with *F* set to zero for negative *F*^2^. The threshold expression of *F*^2^ > 2σ(*F*^2^) is used only for calculating *R*-factors(gt) *etc*. and is not relevant to the choice of reflections for refinement. *R*-factors based on *F*^2^ are statistically about twice as large as those based on *F*, and *R*- factors based on ALL data will be even larger.

### Fractional atomic coordinates and isotropic or equivalent isotropic displacement parameters (Å^2^)

**Table:**

	*x*	*y*	*z*	*U*_iso_*/*U*_eq_	
Br1	0.54911 (3)	0.40086 (2)	0.211746 (16)	0.02854 (12)	
F1	0.52166 (15)	0.13977 (15)	0.52612 (9)	0.0308 (4)	
F2	0.31173 (16)	0.13286 (16)	0.58691 (9)	0.0335 (4)	
F3	0.09951 (16)	0.16152 (16)	0.51060 (10)	0.0364 (5)	
F4	0.08838 (15)	0.18422 (15)	0.36791 (9)	0.0302 (4)	
F5	0.05945 (15)	0.06664 (17)	0.12934 (10)	0.0365 (4)	
F6	0.04016 (16)	−0.11128 (16)	0.02786 (11)	0.0411 (5)	
F7	0.23599 (17)	−0.19317 (16)	−0.02953 (10)	0.0401 (5)	
F8	0.45774 (15)	−0.11084 (14)	0.01579 (9)	0.0282 (4)	
F9	0.85617 (15)	−0.07367 (15)	0.13742 (10)	0.0326 (4)	
F10	1.04672 (16)	−0.14952 (18)	0.22740 (11)	0.0444 (5)	
F11	1.06140 (16)	−0.08814 (17)	0.36440 (11)	0.0442 (5)	
F12	0.88979 (15)	0.05387 (16)	0.41661 (9)	0.0352 (4)	
N1	0.4472 (2)	0.2141 (2)	0.30191 (12)	0.0218 (5)	
N2	0.2545 (2)	0.1752 (2)	0.24173 (12)	0.0231 (5)	
N3	0.4328 (2)	0.1592 (2)	0.18160 (13)	0.0217 (5)	
N4	0.5946 (2)	0.0451 (2)	0.13988 (13)	0.0231 (5)	
N5	0.6199 (2)	0.1505 (2)	0.24953 (12)	0.0213 (5)	
N6	0.6238 (2)	0.1572 (2)	0.37443 (13)	0.0239 (6)	
C1	0.5068 (3)	0.1882 (2)	0.36615 (16)	0.0235 (6)	
C2	0.4140 (3)	0.1777 (2)	0.41463 (15)	0.0221 (6)	
C3	0.4175 (3)	0.1550 (3)	0.48641 (16)	0.0243 (7)	
C4	0.3113 (3)	0.1491 (3)	0.51729 (16)	0.0273 (7)	
C5	0.2002 (3)	0.1616 (3)	0.47737 (17)	0.0271 (7)	
C6	0.1951 (3)	0.1764 (2)	0.40552 (16)	0.0244 (7)	
C7	0.3015 (3)	0.1865 (2)	0.37388 (16)	0.0235 (6)	
C8	0.3254 (3)	0.1999 (2)	0.30061 (16)	0.0228 (6)	
C9	0.3109 (3)	0.1472 (2)	0.18449 (15)	0.0220 (6)	
C10	0.2697 (3)	0.0688 (3)	0.12508 (16)	0.0235 (6)	
C11	0.1577 (3)	0.0248 (3)	0.10206 (16)	0.0283 (7)	
C12	0.1472 (3)	−0.0642 (3)	0.05019 (17)	0.0313 (7)	
C13	0.2494 (3)	−0.1071 (3)	0.02084 (16)	0.0278 (7)	
C14	0.3619 (3)	−0.0637 (3)	0.04319 (15)	0.0249 (7)	
C15	0.3744 (3)	0.0259 (2)	0.09517 (15)	0.0226 (6)	
C16	0.4785 (3)	0.0802 (2)	0.13529 (15)	0.0222 (6)	
C17	0.6618 (3)	0.0783 (2)	0.19883 (16)	0.0220 (6)	
C18	0.7745 (3)	0.0277 (3)	0.23143 (16)	0.0247 (7)	
C19	0.8630 (3)	−0.0444 (3)	0.20584 (17)	0.0276 (7)	
C20	0.9576 (3)	−0.0830 (3)	0.25135 (19)	0.0326 (8)	
C21	0.9660 (3)	−0.0509 (3)	0.32254 (19)	0.0324 (8)	
C22	0.8793 (3)	0.0217 (3)	0.34902 (17)	0.0289 (7)	
C23	0.7829 (3)	0.0615 (3)	0.30424 (16)	0.0238 (6)	
C24	0.6767 (3)	0.1334 (2)	0.31523 (15)	0.0217 (6)	
B1	0.5091 (3)	0.2236 (3)	0.23690 (17)	0.0218 (7)	

### Atomic displacement parameters (Å^2^)

**Table:**

	*U*^11^	*U*^22^	*U*^33^	*U*^12^	*U*^13^	*U*^23^
Br1	0.0370 (2)	0.02078 (17)	0.02777 (19)	−0.00196 (12)	0.00258 (14)	0.00042 (12)
F1	0.0324 (10)	0.0365 (9)	0.0229 (10)	−0.0030 (8)	−0.0018 (8)	0.0011 (7)
F2	0.0411 (11)	0.0404 (10)	0.0193 (10)	−0.0054 (8)	0.0048 (8)	0.0015 (8)
F3	0.0329 (11)	0.0457 (11)	0.0321 (11)	0.0009 (8)	0.0110 (9)	0.0040 (8)
F4	0.0243 (9)	0.0354 (9)	0.0303 (11)	−0.0009 (7)	0.0001 (8)	0.0015 (8)
F5	0.0243 (10)	0.0496 (11)	0.0353 (12)	−0.0035 (8)	0.0017 (8)	−0.0064 (8)
F6	0.0304 (11)	0.0470 (11)	0.0444 (13)	−0.0118 (8)	−0.0053 (9)	−0.0094 (9)
F7	0.0447 (12)	0.0381 (10)	0.0365 (12)	−0.0062 (9)	−0.0024 (9)	−0.0156 (8)
F8	0.0346 (10)	0.0264 (9)	0.0236 (10)	0.0017 (7)	0.0036 (8)	−0.0040 (7)
F9	0.0291 (10)	0.0351 (10)	0.0338 (11)	0.0012 (8)	0.0037 (8)	−0.0094 (8)
F10	0.0286 (11)	0.0462 (11)	0.0576 (14)	0.0126 (9)	−0.0004 (10)	−0.0105 (10)
F11	0.0279 (11)	0.0512 (12)	0.0510 (14)	0.0107 (8)	−0.0102 (9)	0.0040 (9)
F12	0.0326 (10)	0.0442 (10)	0.0273 (11)	−0.0021 (8)	−0.0053 (8)	0.0045 (8)
N1	0.0225 (13)	0.0217 (12)	0.0210 (14)	0.0004 (10)	0.0008 (11)	−0.0024 (10)
N2	0.0247 (13)	0.0254 (12)	0.0188 (14)	0.0037 (10)	−0.0004 (11)	−0.0002 (10)
N3	0.0232 (13)	0.0215 (12)	0.0202 (14)	0.0014 (10)	0.0006 (10)	0.0022 (10)
N4	0.0248 (14)	0.0243 (12)	0.0203 (14)	−0.0022 (10)	0.0023 (11)	0.0015 (10)
N5	0.0246 (13)	0.0202 (12)	0.0189 (14)	−0.0022 (10)	0.0012 (11)	−0.0009 (10)
N6	0.0256 (14)	0.0233 (12)	0.0222 (14)	−0.0037 (11)	−0.0020 (11)	−0.0007 (10)
C1	0.0260 (16)	0.0182 (14)	0.0255 (17)	−0.0012 (12)	−0.0028 (13)	−0.0034 (12)
C2	0.0242 (16)	0.0216 (14)	0.0199 (16)	−0.0020 (12)	−0.0012 (13)	−0.0027 (11)
C3	0.0282 (17)	0.0226 (15)	0.0214 (17)	−0.0018 (13)	−0.0015 (13)	−0.0011 (12)
C4	0.0376 (19)	0.0230 (15)	0.0214 (17)	−0.0028 (14)	0.0037 (14)	−0.0005 (12)
C5	0.0289 (18)	0.0252 (16)	0.0283 (19)	0.0019 (13)	0.0099 (14)	0.0014 (13)
C6	0.0230 (16)	0.0222 (14)	0.0278 (18)	−0.0021 (12)	0.0016 (13)	−0.0003 (12)
C7	0.0285 (17)	0.0196 (14)	0.0222 (17)	−0.0010 (12)	0.0018 (13)	−0.0006 (12)
C8	0.0242 (16)	0.0209 (14)	0.0233 (17)	0.0034 (12)	0.0014 (13)	−0.0001 (12)
C9	0.0238 (16)	0.0194 (14)	0.0219 (17)	0.0013 (12)	−0.0032 (13)	0.0036 (12)
C10	0.0266 (16)	0.0231 (14)	0.0203 (16)	−0.0016 (13)	−0.0008 (13)	0.0003 (12)
C11	0.0257 (17)	0.0353 (17)	0.0236 (17)	−0.0001 (14)	0.0013 (14)	0.0004 (13)
C12	0.0256 (17)	0.0362 (17)	0.0303 (19)	−0.0081 (14)	−0.0071 (14)	0.0009 (14)
C13	0.0371 (19)	0.0246 (15)	0.0205 (17)	−0.0043 (13)	−0.0031 (14)	−0.0041 (12)
C14	0.0323 (18)	0.0235 (15)	0.0191 (16)	0.0017 (13)	0.0029 (14)	0.0008 (12)
C15	0.0273 (16)	0.0220 (14)	0.0181 (16)	0.0010 (12)	0.0002 (13)	0.0045 (11)
C16	0.0266 (16)	0.0218 (14)	0.0178 (16)	−0.0029 (12)	0.0005 (12)	0.0016 (11)
C17	0.0235 (16)	0.0219 (14)	0.0212 (16)	−0.0039 (12)	0.0047 (13)	0.0008 (12)
C18	0.0221 (16)	0.0219 (14)	0.0297 (18)	−0.0029 (12)	0.0003 (13)	0.0009 (12)
C19	0.0267 (17)	0.0234 (15)	0.033 (2)	−0.0043 (13)	0.0036 (14)	−0.0020 (13)
C20	0.0219 (17)	0.0312 (17)	0.045 (2)	0.0007 (14)	0.0031 (15)	−0.0010 (15)
C21	0.0225 (17)	0.0321 (17)	0.040 (2)	0.0007 (14)	−0.0077 (15)	0.0032 (15)
C22	0.0286 (18)	0.0299 (16)	0.0277 (19)	−0.0057 (14)	−0.0012 (14)	0.0029 (13)
C23	0.0212 (16)	0.0244 (14)	0.0257 (17)	−0.0030 (12)	0.0023 (13)	0.0040 (12)
C24	0.0237 (16)	0.0203 (14)	0.0205 (17)	−0.0050 (12)	−0.0012 (13)	0.0004 (12)
B1	0.0264 (18)	0.0197 (16)	0.0189 (18)	−0.0035 (14)	−0.0007 (15)	0.0001 (13)

### Geometric parameters (Å, °)

**Table:**

Br1—B1	2.047 (3)	N6—C1	1.345 (4)
F1—C3	1.339 (3)	N6—C24	1.346 (4)
F2—C4	1.339 (3)	C1—C2	1.454 (4)
F3—C5	1.340 (3)	C2—C3	1.388 (4)
F4—C6	1.336 (3)	C2—C7	1.419 (4)
F5—C11	1.337 (3)	C3—C4	1.373 (4)
F6—C12	1.334 (3)	C4—C5	1.402 (4)
F7—C13	1.340 (3)	C5—C6	1.376 (4)
F8—C14	1.336 (3)	C6—C7	1.385 (4)
F9—C19	1.338 (4)	C7—C8	1.454 (4)
F10—C20	1.344 (4)	C9—C10	1.459 (4)
F11—C21	1.335 (4)	C10—C11	1.373 (4)
F12—C22	1.330 (3)	C10—C15	1.425 (4)
N1—C8	1.366 (4)	C11—C12	1.381 (4)
N1—C1	1.369 (4)	C12—C13	1.397 (4)
N1—B1	1.477 (4)	C13—C14	1.373 (4)
N2—C8	1.341 (4)	C14—C15	1.388 (4)
N2—C9	1.344 (4)	C15—C16	1.458 (4)
N3—C16	1.365 (4)	C17—C18	1.460 (4)
N3—C9	1.373 (4)	C18—C19	1.385 (4)
N3—B1	1.472 (4)	C18—C23	1.431 (4)
N4—C17	1.343 (4)	C19—C20	1.371 (4)
N4—C16	1.347 (4)	C20—C21	1.396 (5)
N5—C17	1.361 (4)	C21—C22	1.381 (4)
N5—C24	1.364 (4)	C22—C23	1.382 (4)
N5—B1	1.472 (4)	C23—C24	1.451 (4)
			
C8—N1—C1	113.3 (2)	F6—C12—C11	120.9 (3)
C8—N1—B1	122.3 (3)	F6—C12—C13	119.0 (3)
C1—N1—B1	122.6 (2)	C11—C12—C13	120.1 (3)
C8—N2—C9	116.1 (2)	F7—C13—C14	120.1 (3)
C16—N3—C9	113.4 (2)	F7—C13—C12	118.6 (3)
C16—N3—B1	122.5 (2)	C14—C13—C12	121.2 (3)
C9—N3—B1	121.9 (2)	F8—C14—C13	119.3 (3)
C17—N4—C16	116.0 (2)	F8—C14—C15	121.1 (3)
C17—N5—C24	114.3 (2)	C13—C14—C15	119.5 (3)
C17—N5—B1	122.3 (2)	C14—C15—C10	118.9 (3)
C24—N5—B1	122.5 (2)	C14—C15—C16	133.1 (3)
C1—N6—C24	116.5 (2)	C10—C15—C16	107.5 (2)
N6—C1—N1	123.0 (3)	N4—C16—N3	123.5 (3)
N6—C1—C2	130.3 (3)	N4—C16—C15	129.5 (3)
N1—C1—C2	105.6 (2)	N3—C16—C15	105.5 (2)
C3—C2—C7	119.7 (3)	N4—C17—N5	123.2 (3)
C3—C2—C1	133.1 (3)	N4—C17—C18	130.5 (3)
C7—C2—C1	107.1 (3)	N5—C17—C18	104.7 (2)
F1—C3—C4	119.4 (3)	C19—C18—C23	120.1 (3)
F1—C3—C2	121.6 (3)	C19—C18—C17	132.8 (3)
C4—C3—C2	118.9 (3)	C23—C18—C17	107.1 (2)
F2—C4—C3	120.4 (3)	F9—C19—C20	121.0 (3)
F2—C4—C5	118.3 (3)	F9—C19—C18	120.0 (3)
C3—C4—C5	121.3 (3)	C20—C19—C18	119.0 (3)
F3—C5—C6	120.7 (3)	F10—C20—C19	120.2 (3)
F3—C5—C4	118.9 (3)	F10—C20—C21	118.6 (3)
C6—C5—C4	120.5 (3)	C19—C20—C21	121.2 (3)
F4—C6—C5	119.8 (3)	F11—C21—C22	120.2 (3)
F4—C6—C7	121.3 (3)	F11—C21—C20	119.0 (3)
C5—C6—C7	118.9 (3)	C22—C21—C20	120.8 (3)
C6—C7—C2	120.6 (3)	F12—C22—C21	120.1 (3)
C6—C7—C8	131.8 (3)	F12—C22—C23	120.7 (3)
C2—C7—C8	107.5 (2)	C21—C22—C23	119.1 (3)
N2—C8—N1	123.3 (3)	C22—C23—C18	119.8 (3)
N2—C8—C7	129.6 (3)	C22—C23—C24	132.9 (3)
N1—C8—C7	105.3 (3)	C18—C23—C24	107.3 (3)
N2—C9—N3	123.4 (3)	N6—C24—N5	122.7 (3)
N2—C9—C10	128.6 (3)	N6—C24—C23	130.7 (3)
N3—C9—C10	105.6 (2)	N5—C24—C23	105.0 (2)
C11—C10—C15	120.9 (3)	N3—B1—N5	106.4 (2)
C11—C10—C9	131.8 (3)	N3—B1—N1	106.4 (2)
C15—C10—C9	106.9 (2)	N5—B1—N1	106.0 (2)
F5—C11—C10	120.9 (3)	N3—B1—Br1	113.9 (2)
F5—C11—C12	119.9 (3)	N5—B1—Br1	110.5 (2)
C10—C11—C12	119.3 (3)	N1—B1—Br1	113.1 (2)
